# Essential Oils From Different Parts of *Piper nigrum* L.: Chemical Composition, Antibacterial, and Antioxidant Activities

**DOI:** 10.1002/fsn3.71205

**Published:** 2025-11-14

**Authors:** Sarifah Nurjanah, Edy Suryadi, Ahmad Thoriq, Nurul Ainina, Efri Mardawati, Muhammad Gilang Ramadhan, Rosmiati Rosmiati, Abd. Wahid Rizaldi Akili, Nandang Permadi, Euis Julaeha

**Affiliations:** ^1^ Department of Agricultural Engineering and Biosystems, Faculty of Agro‐Industrial Technology Universitas Padjadjaran Bandung West Java Indonesia; ^2^ Department of Agro‐Industrial Technology, Faculty of Agro‐Industrial Technology Universitas Padjadjaran Bandung West Java Indonesia; ^3^ Agroindustry Study Program Subang State Polytechnic Subang Indonesia; ^4^ Research Center for Fisheries‐National Research and Innovation Agency of Central Jakarta Jakarta Indonesia; ^5^ Department of Chemistry, Faculty of Mathematics and Natural Sciences Universitas Padjadjaran Bandung West Java Indonesia; ^6^ Doctorate Program in Biotechnology, Graduate School Universitas Padjadjaran Bandung West Java Indonesia

**Keywords:** antibacterial activity, chemical composition, essential oil, *Piper nigrum* L.

## Abstract

Pepper ( *Piper nigrum* L.) is a spice plant that contains bioactive compounds, including essential oils, with antioxidant and antimicrobial activity. The study intended to determine the composition of essential oil in the pepper plant's seeds, stems, as well as leaves and whether these materials had any potential antioxidant activity, and antibacterial activity against *Escherichia coli* and *Staphylococcus aureus* . GC–MS (Gas Chromatography–Mass Spectrometry) was utilized to investigate the components of pepper oil, and the inhibition diameter was used to determine the antibacterial activity. The findings revealed that 19, 19, and 29 compounds were found in pepper seed, leaf and stem essential oils, respectively. The three most important contents of seed essential oil were *δ*‐3‐carene (11.49%), limonene (13.35%), and *β*‐caryophyllene (37.42%). Leaf essential oil contained *δ*‐elemene (3.73%), *δ*‐3‐carene (19.03%), and *β*‐caryophyllene (50.50%), while stem essential oil was dominated by *α*‐selinene (11.93%), *β*‐caryophyllene (12.83%), and *δ*‐elemene (19.73%). Principal Component Analysis (PCA) revealed clear compositional differences among the three essential oils. Antibacterial assays showed variable activity, with inhibition zones against *E. coli* measuring 1.5 mm, 6.83 mm, and 4.83 mm, for seed, leaf, and stem oils, respectively, and against *S. aureus* measuring 8.89 mm, 8.06 mm, and 13.00 mm. Antioxidant activity, evaluated by the DPPH assay demonstrated that stem essential oil exhibited the strongest radical scavenging effect (IC_50_ 255.10 ppm; TEAC 8.39 μmol TE/g), followed by leaf oil (IC_50_ 358.62 ppm; TEAC 5.98 μmol TE/g) and seed oil (IC_50_ 485.98 ppm; TEAC 4.41 μmol TE/g), though all were considered weak antioxidants. These results indicate that *P. nigrum* essential oils vary significantly in chemical composition and bioactivity across plant parts. The findings confirm their antibacterial and antioxidant potential and underscore the value of seeds, leaves, and stems as alternative sources of bioactive essential oils for prospective applications in food preservation, pharmaceuticals, and natural health products.

## Graphical abstract

This study investigates the essential oil composition from the seeds, stems, and leaves of *Piper nigrum* L. and evaluates their antibacterial activity against Escherichia coli and Staphylococcus aureus. GC‐MS analysis identified key compounds such as *β*‐caryophyllene, *δ*‐3‐carene, and limonene, while antibacterial testing showed promising inhibition zones, particularly against *S. aureus*. The results suggest that black pepper essential oil holds potential as a natural antibacterial agent.

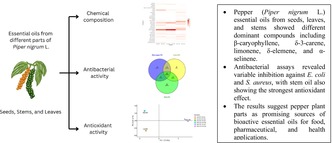

## 1 Introduction

Essential oils are volatile oils and have a specific aroma according to the plant of origin, commonly extracted by distillation processes (Shehata et al. 2020). They have been widely used in the food, cosmetic, pharmaceutical, and chemical industries (Cui et al. 2019; Ni et al. 2021). Essential oils have good biological activity, including antioxidant, antiviral, antifungal, and antibacterial properties (Hasheminejad and Khodaiyan 2020; Morshdy et al. 2021; Yang et al. 2021). Monoterpenes, oxygen‐containing terpene hydrocarbons, aldehydes, flavonoids, alkaloids, phenolic acids, isoflavones, and other aromatic compounds make up essential oils (Gedikoğlu et al. 2019; Ni et al. 2021). Essential oils are secondary metabolites typically present in several plant parts, such as the roots, trunks, bark, stems, leaves, flowers, and fruits (Cui et al. 2019). Numerous variables, including physicochemical characteristics, soil composition, solar exposure, geographic location, and the plant section used, might affect the concentration of these secondary metabolites (Gharehbagh et al. 2023; Lee et al. 2020).

Pepper ( *Piper nigrum* L.) is a tropical crop of the Piperaceae family, a common spice widely distributed in Asia (India, Vietnam, and Indonesia). Pepper is used mainly as a flavoring agent in foods, with extensive potential in applications for nutraceutical and ethnomedicine (Heckert Bastos et al. 2020). The black pepper essential oil or its extract exhibits antifungal, antioxidant, antiamoebic, antiasthmatic, antidiabetic, and immunomodulatory activities. In comparison, white pepper has many functions, such as a preservative, antibacterial, antifungal, antiprotozoal, and insecticide (Lee et al. 2020).

Essential oil from black pepper showed antimicrobial activity against *Pseudomonas aeruginosa, Salmonella typhimurium*, and *Listeria monocytogenes* (Rodrigues dos Santos et al. 2023) and foodborne pathogens of *Salmonella* sp., *Escherichia coli* , *Listeria* sp., and *Staphylococcus aureus* (Milagres de Almeida et al. 2023). *S. aureus* is a pathogenic bacterium that easily contaminates high‐protein foods such as eggs, fish, and milk. Food poisoning due to the presence of Staphylococcal is very common in humans and animals (dos Santos Rodrigues et al. 2017; Kang et al. 2019; Wang et al. 2020). *E. coli* , in addition to being a pathogenic bacterium that poisons food, can cause complications in human health (Guo et al. 2020; Wang et al. 2020).

In addition to their antimicrobial properties, essential oils are also recognized for their antioxidant activity, which plays a vital role in preventing oxidative stress and related degenerative diseases. Antioxidants in essential oils act primarily through free radical scavenging and metal‐chelating mechanisms, thereby contributing to food preservation and human health (Tit and Bungau 2023). Several studies have reported that the antioxidant potential of essential oils is closely associated with their chemical composition, especially the presence of phenolic compounds, sesquiterpenes, and oxygenated monoterpenes (Kusumorini et al. 2022; Lee et al. 2020). In the case of *P. nigrum* , sesquiterpene‐rich fractions such as *β*‐caryophyllene, *δ*‐elemene, and *α*‐selinene have been linked with moderate antioxidant activity, although the strength varies depending on plant parts and extraction conditions (Kusumorini et al. 2022; Morshdy et al. 2021). These findings underscore the importance of exploring pepper essential oils not only for their antibacterial effects but also for their antioxidant potential, which enhances their prospective applications in food preservation, nutraceuticals, and pharmaceuticals.

Recent studies have further expanded this knowledge. Black pepper essential oil has been shown through in silico analyses to interact strongly with bacterial proteins such as FtsZ, GyrB, murA, and PTP, supporting its antibacterial potential (Farida et al. 2025). Off‐grade white pepper essential oils obtained by microwave‐assisted hydro‐distillation exhibited significant antibacterial activity, indicating the value of otherwise wasted material (Nurjanah et al. 2025). In addition, notable antimicrobial and antibiofilm effects of black pepper essential oil have been reported both in vitro and in situ, highlighting its promise as a natural preservative in food safety (Vuković et al. 2024).

The main components of pepper essential oil were monoterpene hydrocarbons (47%–64%) and sesquiterpene hydrocarbons (30%–47%) (Weluwanarak et al. 2023). The volatile flavoring chemicals *β*‐pinene, limonene, *δ*‐3‐carene, and caryophyllene are widely present in white, black, and green peppers. More *α*‐pinene and *α*‐copaene were found in the pericarp than in the pepper seed. Terpenic odor is brought on by *α*‐pinene. As a result, the pericarp smells more like terpenes than black, white, or green pepper (Lee et al. 2020). Moreover, essential oil from green pepper had the highest level of oxygenated terpenoids such as eugenol, hedycaryol, caryophyllene oxide, and *β*‐eudesmol, and sesquiterpenes such as *β*‐caryophyllene when compared with white and black pepper (Weluwanarak et al. 2023).

Indonesia is the second largest pepper producer after Vietnam, producing 83,000 tons in 2021. Pepper cultivation is spread in almost all provinces, with Bangka‐Belitung province being the most extensive area of 49 ha with an amount of production of 27,000 tons (Sekretariat Jenderal‐Kementerian Pertanian 2022). Pepper is processed into two products, namely white and black pepper. The difference between white and black pepper lies in their processing techniques. White pepper is obtained by removing the pericarp through soaking in water before drying, whereas black pepper is produced by directly drying the harvested pepper seeds.

Although the chemical composition of black pepper, white pepper, green pepper, and pepper pericarp has been studied (Lee et al. 2020; Weluwanarak et al. 2023), no research has yet been done on the stems and leaves of pepper from Muntok, Bangka Belitung, Indonesia. The plants, including stems and leaves, are often removed when old enough and unable to generate seed anymore. It would be profitable if the stems and leaves could be used to make essential oil. The present study aimed to identify the chemical components and antibacterial activity of essential oil from stems, leaves, and black pepper from Muntok, against *E. coli* and *S. aureus* .

## 2 Materials and Method

### 2.1 Materials

The material used in this study were *P. nigrum* stems, leaves, and black pepper seed, which were grown, harvested, and processed in Muntok, Bangka Belitung, Indonesia. Stems and leaves were collected and dried at room temperature, away from sunlight. Pepper seeds were picked, dried, and processed to grind and ready to extract of the essential oils. *S. aureus* and *E. coli* were supplied by the Food Microbiology Laboratory, Universitas Padjadjaran (UNPAD). All chemical reagents made by E‐Merck, Germany, Nutrient Agar (NA), and Mueller‐Hinton Agar (MHA) (PA, Oxoid, England).

### 2.2 Pepper Essential Oil Extraction

The essential oils were extracted by hydro‐distillation for 4 h (Mehta et al. 2023). Next, the essential oil was collected into a vial with anhydrous sodium sulfate to remove any water. Then, vials were stored at 4°C for later analysis. The essential oils yield is expressed in % (v/w) based on the fresh‐plant material weight.

### 2.3 Chemical Composition by Gas Chromatography–Mass Spectrometry (GC–MS)

GC–MS analysis was performed using an Agilent 7890 gas chromatograph equipped with an HP‐INNOWAX capillary column (30 m × 0.25 mm, film thickness 0.25 μm) and an Agilent 5975A mass spectrometer. 1 μL of diluted samples (with ethyl acetate) was injected into the injection site. The temperature program was scheduled as follows: the initial temperature of the oven was 60°C hold for 0 min, rising at 2°C/min to 150°C hold for 1 min, and finally rising at 20°C/min to 210°C hold for 10 min. Helium was used as a carrier gas with a 0.6 mL/min flow rate. The ionization voltage of the detector was set at 70 eV. Essential oil components are identified by comparing them with the n‐alkane series (C8–C30) under the same conditions. Identification of LPO components was carried out using retention indices (RI) via the NIST 2.0 library version.

### 2.4 Antibacterial Activity of Pepper Essential Oils

*S. aureus* and *E. coli* were prepared with cell content 3 × 10^8^ CFU mL^−1^ (absorption in spectrometry was compared with the Mc. Farland). *E. coli* and *S. aureus* were grown on NA medium. The suspensions were dispersed on MHA plates using a moist, sterile swab. One microliter of essential oil was poured into the well and then incubated for 48 h. The diameter of inhibition was evaluated following incubation to ascertain the antibacterial activity. The widths of the inhibitory zones resulting from this comparison contrasted with the control without any essential oils.

### 2.5 Antioxidant Activity of Pepper Essential Oils

The antioxidant activity of the samples was evaluated following the procedures of Kusumorini et al. (2022) and Widyaningrum et al. (2025). In brief, 2 mL of 0.1 mM DPPH (2,2‐diphenyl‐1‐picrylhydrazyl) solution was mixed with 2.0 mL of the test sample at concentrations ranging from 100 to 1000 ppm. The mixtures were incubated at room temperature (±25°C) for 30 min in the dark, after which the absorbance was measured at 517 nm using a UV–VIS spectrophotometer. All assays were performed in triplicate. The free radical scavenging activity was calculated by comparing the absorbance of the test solution with that of the control (containing only DPPH and ethanol), and the percentage inhibition was determined using Equation:

$$\%\text{Inhibition}=\frac{\mathrm{Abs}\;\text{Control}-\mathrm{Abs}\;\text{Sample}}{\mathrm{Abs}\;\text{Control}}\times 100\%$$

The IC_50_ values were obtained from linear regression analysis of the inhibition data. Trolox was employed as a positive control, and a standard calibration curve was constructed to express the antioxidant capacity of the samples as Trolox equivalent antioxidant capacity (TEAC) (Adedayo et al. 2020).

### 2.6 Statistical Analysis

To determine the mean, standard deviation, and analysis of variance (ANOVA) of the test results (tree replication), Microsoft Excel for Mac ver 16.78.3 (Analysis Tools) software was used.

## 3 Results

### 3.1 GC–MS Analysis of Pepper Essential Oil

**FIGURE 1:**
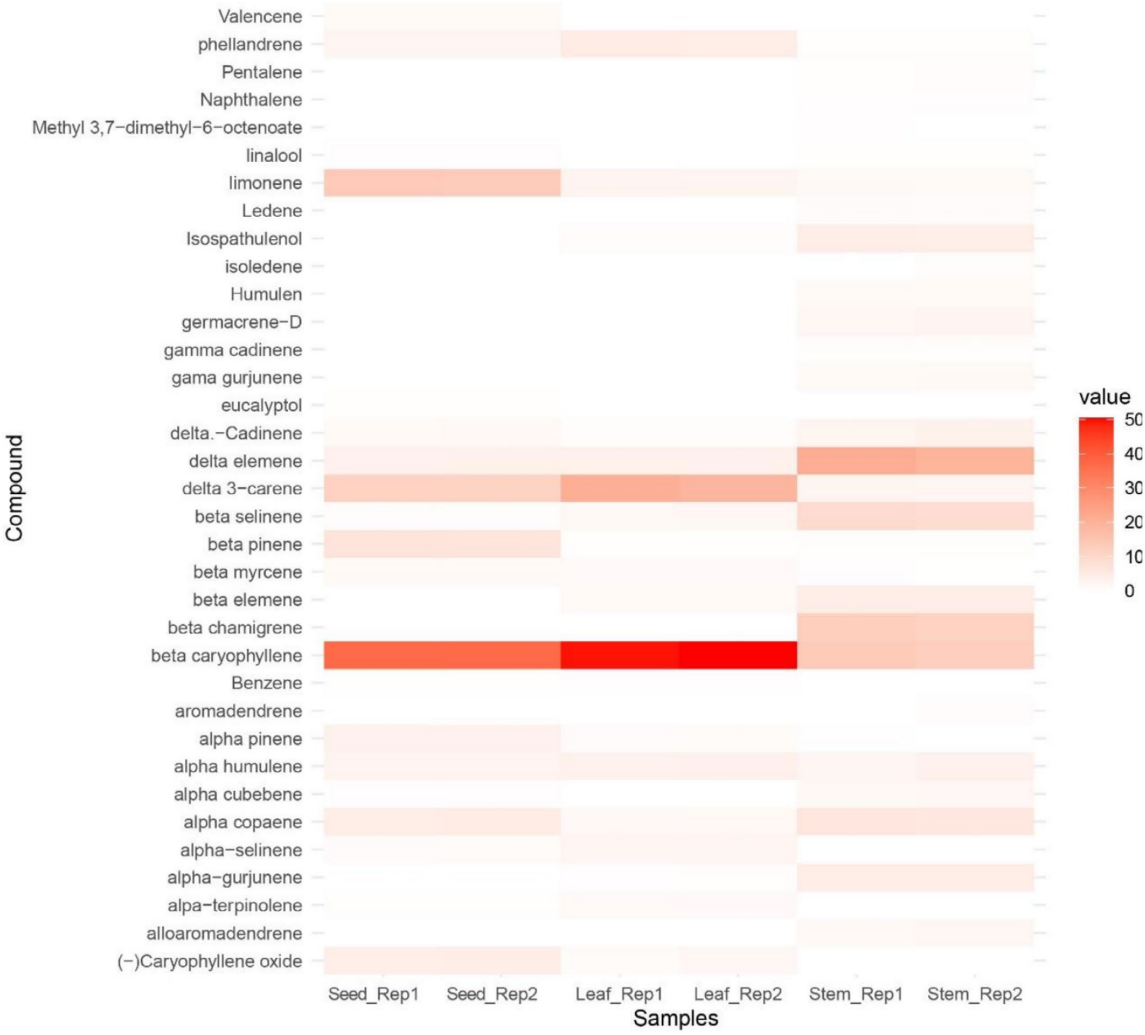
Heat map of relative volatile component content of black pepper seeds, stem, and leaves oils of *P. nigrum*.

**FIGURE 2:**
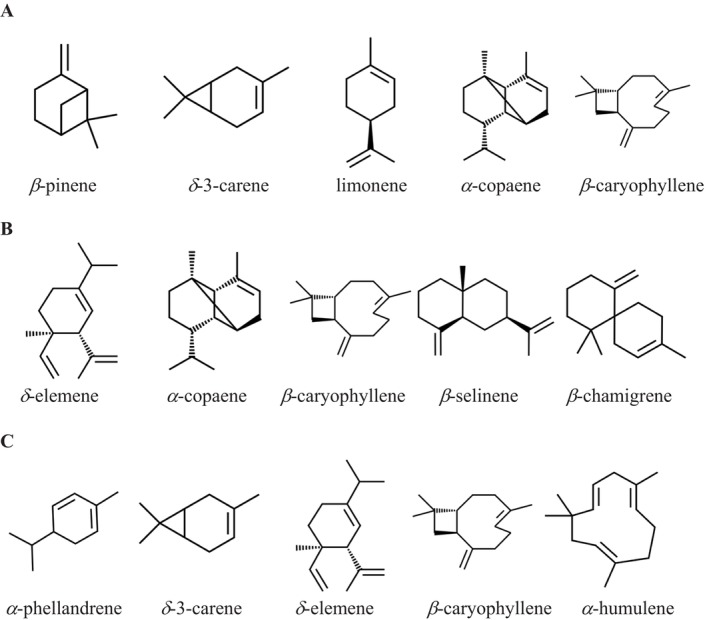
Major components of black pepper seeds (A), stems (B), and leaves (C) oil of *P. nigrum*.

The GC–MS results of black pepper, stem, and leaf oil of *P. nigrum* were presented as a heat map shown in Figure 1. In the black pepper seeds oil, 20 compounds were identified in which 40.93% were monoterpenes hydrocarbons, 0.80% were oxygenated monoterpenes, 53.79% sesquiterpenes hydrocarbons, and 4.48% were oxygenated hydrocarbons. In the stems oil, 29 compounds were identified, of which 5.67% were monoterpenes hydrocarbons, 0.29% were oxygenated monoterpenes, 88.86% were sesquiterpenes hydrocarbons, 5.06% were oxygenated hydrocarbons, and 0.18% was another compound. In the leaves oil, 19 compounds were identified, of which 31.86% were monoterpenes hydrocarbons, no oxygenated monoterpenes, 66.30% were sesquiterpenes hydrocarbons, and 1.83% were oxygenated hydrocarbons. Five major components of each essential oil are presented in Figure 2.

**FIGURE 3:**
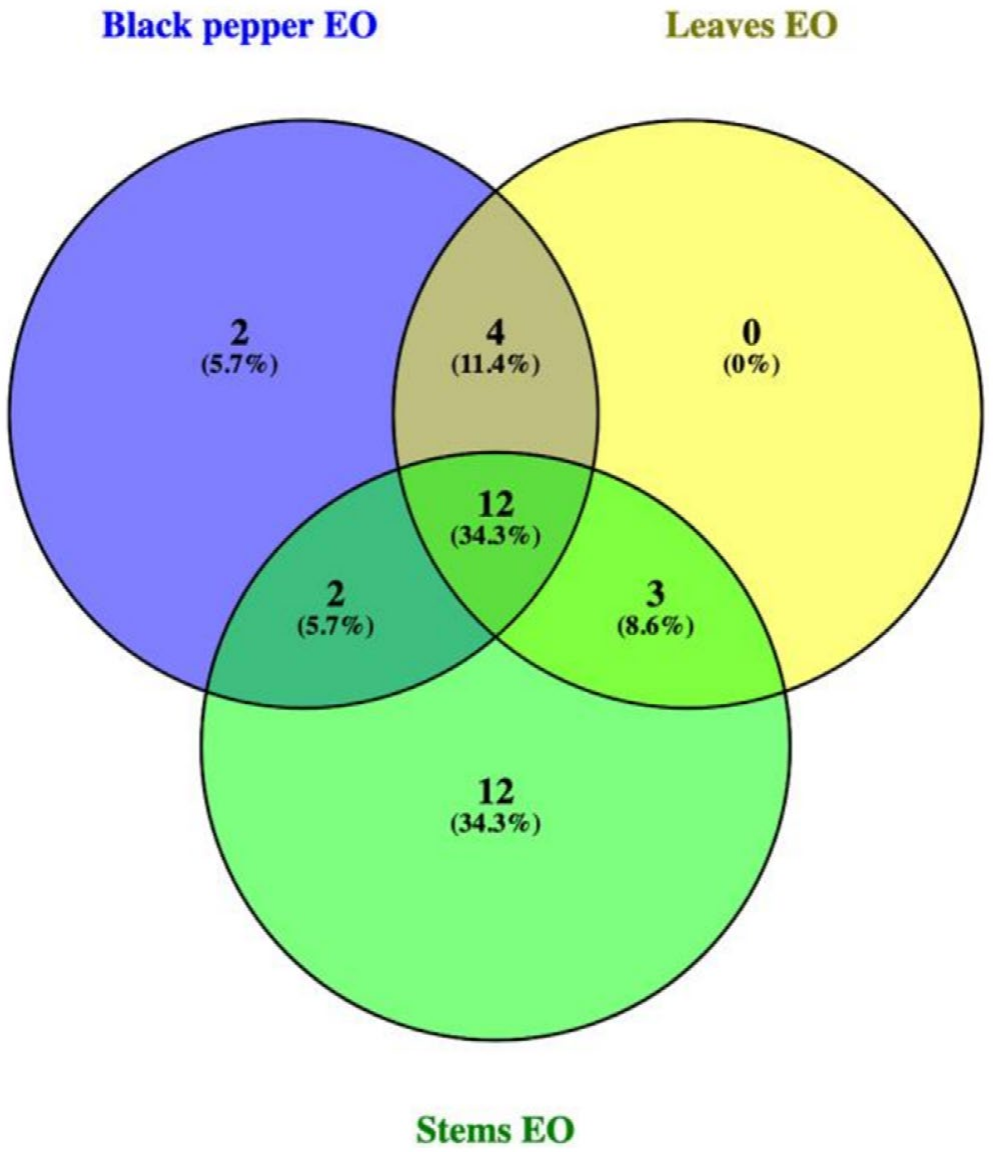
Venn diagram of black pepper seeds (A), stems (B), and leaves (C) oil of *P. nigrum*.

The results showed that the three essential oils had different components. However, there were similar components for *α*‐pinene, *β*‐pinene, *β*‐myrcene, phellandrene, *δ*‐3‐carene, limonene, *δ*‐element, *α*‐copaene, *β*‐caryophyllene, *α*‐humulene, *β*‐selinene, and *δ*‐cadinene (Figure 3). Among the three types of essential oils, the essential oil components of leaves were partly contained in black pepper essential oil and stem essential oil. In contrast, black pepper and stem essential oils had some unique components not found in essential oils from other parts. Components found only in the black pepper seeds were eucalyptol and valencene, while components that were only found in stems essential oil were methyl 3,7‐dimethyl‐6‐octenoate, aromadendrene, isoledene, *γ*‐gurjunene, alloaromadendrene, germacrene‐D, *β*‐chamigrene, *γ*‐cadinene, naphthalene, humulene, ledene, and pentalene.

### 3.2 Principal Component Analysis

Principal component analysis (PCA) is a statistical technique commonly used for reducing the dimensionality of large datasets while preserving as much variability as possible. It transforms the original variables into a new set of orthogonal variables called principal components (PCs), which are ordered such that the first few components explain the most variance in the data. This technique is especially useful for visualizing and interpreting complex datasets with many variables, allowing researchers to identify patterns, clusters, or trends that may not be apparent in higher‐dimensional space (Pramitha et al. 2025). In our research, we employed PCA utilized to investigate the similarities and differences in the chemical composition of essential oils derived from three distinct plant parts: seeds, leaves, and stems of *P. nigrum* .

**FIGURE 4:**
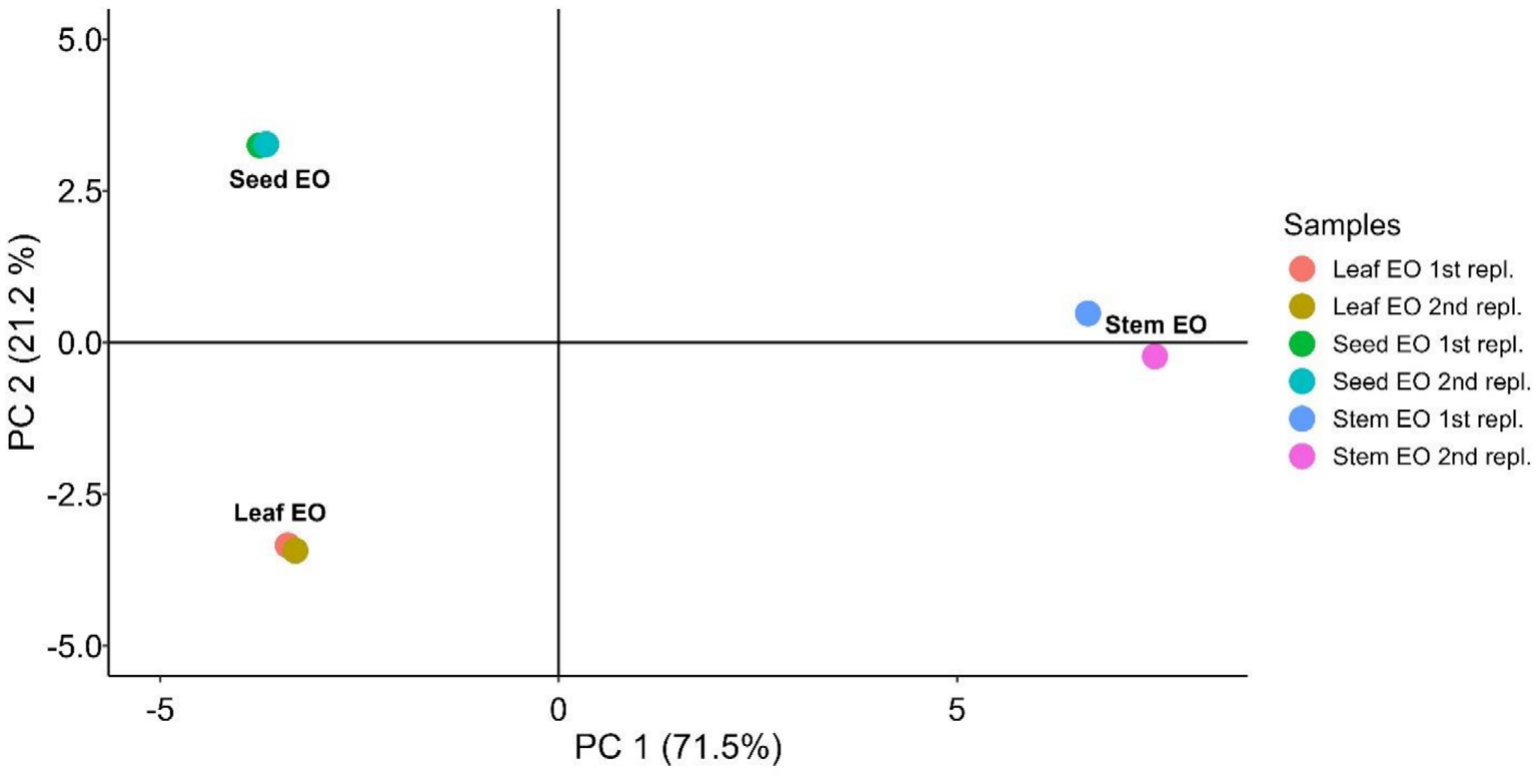
Score plot of seed, leaf, and stem essential oil in PC 1 and PC 2.

From the results of PCA conducted on the relative abundance of volatile compounds identified by GC–MS for each sample, we found that the first PC accounts for 71.5% of the variability in the data, while the second PC accounts for 21.2%. Together, the first two PCs explain more than 90% of the total data variability. The score plot of the leaf, seed, and stem essential oils (EOs) in these two PCs reveals that the data points are distinctly separated (Figure 4), highlighting their unique EO profiles. Interestingly, the positioning of samples in PC 1 demonstrates that both leaf and seed samples exhibit negative scores, whereas stem samples display positive scores. This suggests that the EOs derived from the leaf and seed share considerable similarities, distinguishing them from the EO obtained from the stem.

### 3.3 Physical Properties of Black Pepper, Stems, and Leaves Oil of *P. nigrum* L.

Table 1 shows the physical properties of the oil of black pepper seed, stems, and leaves of *P. nigrum* . The color differed between seed, leaf, and stem essential oils. The density and refractive index also showed different values, while the appearance, odor, and solubility in ethanol were the same.

**TABLE 1:** The physical and chemical characteristics of black pepper, stems, and leaves essential oil of *P. nigrum* L.

Sample	Appearance	Color	Odor	Density (g/mL)	Refractive index (20°C)	Optical rotation (^o^)	Solubility on alcohol (95% at 20°C)
Seed EO	Clear liquid	Yellow	Pepper like	0.860	1.564	−7.48	1:1
Leaf EO	Clear liquid	Dark greenish yellow	Pepper like	0.890	1.582	N/A	1:1
Stem EO	Clear liquid	Greenish yellow	Pepper like	0.880	1.567	N/A	1:1

### 3.4 Antimicrobial Activity of Essential Oil

In inhibition zones of black pepper, stems, and leaves essential oil of *P. nigrum* against the two bacteria was shown in Table 2, which showed antibacterial on *S. aureus* and *E. coli* .

**TABLE 2:** Inhibition zones of seed (black pepper), stems, and leaves essential oil of *P. nigrum* .

Sample	Inhibition zones (mm)	
	*S. aureus*	*E. coli*
Seed EO	8.75b*	1.58a*
Leaf EO	7.58a*	6.83a**
Stem EO	13.00b**	4.83a**

*Note:* Different letters within the same row indicate significant differences at *p* < 0.05. Symbols * and ** within the same column indicate significant differences at *p* < 0.05.

### 3.5 Antioxidant Activity of Pepper Essential Oils

The antioxidant activity of each sample was evaluated in triplicate, and the IC_50_ values obtained from the DPPH assay are shown in Table 3. These values were then converted to TEAC, which expresses the antioxidant strength of the samples relative to the standard antioxidant Trolox.

**TABLE 3:** Antioxidant activity of pepper essential oils expressed as IC_50_ (DPPH assay) and TEAC values.

Sample	IC_50_ (ppm)	TEAC (μmol TE/g)
Seed EO	485.98a	4.41a
Leaf EO	358.62b	5.98b
Stem EO	255.10c	8.39c

*Note:* Different letters within the same column indicate significant differences at *p* < 0.05.

## 4 Discussion

### 4.1 GC–MS Analysis of Pepper Essential Oil

Previous studies reported that constituents of essential oil from seed, leaf, and stem of *P. nigrum* were varied (Salehi et al. 2019; Xiang et al. 2017) and were confirmed with the present study. Among the three essential oils, stem essential oil contained a higher number of components, which was 12 components that were not found in the black pepper seed and leaves essential oils. However, all essential oils had the odor of pepper because *α*‐pinene, myrcene, phellandrene, and limonene as determinants of pepper oil aroma (Lee et al. 2020; Mamatha et al. 2008) were presented in black pepper seeds, stems and leaves essential oils studied, even though in different amount. Black pepper seeds essential oil contained more *α*‐pinene than stem and leaves essential oils. *α*‐Pinene emits a terpenic odor (Murthy and Bhattacharya 2008). As a result, black pepper essential oil smelled more terpenic than stem and leaves essential oil.

In our study, we found that *δ*‐3‐carene (11.49%), limonene (13.35%), and *β*‐caryophyllene (37.42%) were major components of black pepper essential oils (Figure 1). Compared to the other black pepper cultivars of previous studies (Morshed et al. 2017; Singh et al. 2004; Tran et al. 2019), the content of *β*‐caryophyllene was significant. While for *δ*‐3‐carene and limonene, the compounds were found in lower quantities than black pepper from Malaysia, India, and Vietnam (Ashokkumar et al. 2021; Lawrence 2010; Tran et al. 2019).

In the present study, sesquiterpene hydrocarbons dominated the composition of black pepper essential oil, followed by monoterpene hydrocarbons and oxygenated monoterpenes. *β*‐caryophyllene, *α*‐copaene, *δ*‐elemene, and *α*‐humulene were the major sesquiterpene hydrocarbon constituents present in black pepper essential oil. The other sesquiterpenes present in substantial amounts were *α*‐humulene, *α*‐selinene, *α*‐cubebene, and valencene. However, valencene was only found in black pepper seeds essential oil and not in stem and leaves essential oils. It is also important to note that Indonesian black pepper oil does not contain sabinene, like black pepper cultivars from Bangladesh and Vietnam (Morshed et al. 2017; Tran et al. 2020). The absence of sabinene may be explained by several factors, including genetic variation among pepper cultivars, agro‐climatic and soil conditions, harvest maturity and post‐harvest processing, as well as methodological differences in oil extraction and GC–MS detection (Permadi et al. 2024). This observation highlights the chemical diversity of black pepper essential oils across different geographical origins. Furthermore, seven terpene hydrocarbons were identified in black pepper essential oil. Among them, limonene was the predominant constituent, followed by *δ*‐3‐carene, *β*‐pinene, *α*‐pinene, phellandrene, and *β*‐myrcene.

In the leaves essential oil of *P. nigrum* , sesquiterpene hydrocarbons were the major composition, followed by monoterpene hydrocarbons and oxygenated sesquiterpenes. It is noted that no oxygenated monoterpene was found. Among the sesquiterpene hydrocarbons, *β*‐caryophyllene was the predominant constituent in the leaf's essential oil. The highest *β*‐caryophyllene content of 50.10% was noticed in the leaf's essential oil, which was about twofold higher than the black pepper seeds essential oil and more than threefold as compared to stem essential oil. Moreover, it was higher than the leaves and stem of pepper essential oils from China (Salehi et al. 2019; Xiang et al. 2017). The other sesquiterpene present in substantial amounts were *δ*‐elemene, *α*‐copaene, *β*‐elemene, *α*‐humulene, and *β*‐cadinene. However, leaves essential oil did not contain *α*‐cubebene and linalool, like black pepper seeds and stem essential oils. Furthermore, seven terpene hydrocarbons were identified; among them, *δ*‐3‐carene was the major component in black pepper seeds essential oil, followed by phellandrene, limonene, *α*‐terpinolene, *β*‐myrcene, and *α*‐pinene.

Pepper stem essential oil presented the most components at 29 constituents compared to leaf and black pepper seed essential oils. However, like leaf and black pepper seed essential oils, sesquiterpene hydrocarbons were the dominant component (88.86%). Among these components, *δ*‐elemene (20.38%) was the most dominant component, followed by *β*‐caryophyllene (13.31%) and *β*‐chamigrene (12.32%). *δ*‐elemene is the sesquiterpene that has proven to have anticancer activity in several previous studies (Al‐Radadi 2022), inhibit bladder cancer cells (Pei et al. 2023), esophageal cancer (Zhou et al. 2023), and lung adenocarcinoma (Song et al. 2023), and revealed antibacterial activity (González et al. 2021). Moreover, stem essential oil contains high *β*‐chemigrene, which is not contained in black pepper seeds and leaves. Earlier studies revealed that *β*‐chemigrene has antibacterial (Bansemir et al. 2004), antifeedant (White et al. 2010), and antifouling properties (Al‐Lhaibi et al. 2015; Dahms and Dobretsov 2017).

### 4.2 Principal Component Analysis

In PCA, the correlation coefficients between the variables and the PCs can be used to determine which variables (in this case, the volatile compounds) most influence each principal component (PC). A high correlation means that the variable has a strong effect on the component's variability (Akili et al. 2023). Table S1 summarizes the variables significantly correlated with PC 1. Notably, compounds such as humulene, germacrene D, ledene, naphthalene, γ‐cadinene, γ‐gurjunene, β‐chamigrene, *δ*‐elemene, alloaromadendrene, *α*‐gurjunene, *β*‐selinene, isospathulenol, *α*‐cubebene, pentalene, *β*‐elemene, and *δ*‐cadinene exhibited positive correlations with PC 1. This indicates that samples with a positive PC 1 score (i.e., stem EO samples) contain higher levels of these metabolites. Conversely, compounds such as *α*‐terpinolene, *δ*‐3‐carene, *β*‐caryophyllene, benzene, and *β*‐myrcene were negatively correlated with PC 1. These metabolites are more abundant in samples with negative PC 1 values, such as those EO derived from seeds and leaves, and less abundant in stem EO samples.

Even though the leaf and seed essential oils shared a negative score in PC 1, suggesting both samples share similar metabolites that correlate to PC 1, the score in PC 2 of these samples is different (Figure 4). Among the variables that correlate to PC 2 are linalool, *β*‐pinene, eucalyptol, and Valencene (Table S2). These compounds have a positive correlation to PC 2, suggesting that these compounds are more abundant in the seed EO and less abundant in the leaf EO, as the seed EO samples have a positive score whereas the leaf EO samples have a negative score in PC 2.

### 4.3 Physical Properties of Pepper Essential Oils

The color showed a difference between seed, leaf, and stem essential oils; the density and refractive index also showed a different value, while the appearance, odor, and solubility in ethanol were the same. The different density and index refraction value is caused by different oil components (Quoc 2021). Relative density was the proportion of the mass of a given volume of the essential oil to the mass of an equal volume of distilled water at 20°C (Bisht et al. 2021).

The present study showed that black pepper seeds essential oil density was lower than that of its leaves and stem. As we can see from the components, the black pepper seeds essential oil contained more monoterpenes than leaves and stem essential oils, while the highest density was found in the stem essential oil due to its high content of sesquiterpenes (88.86%). ISO 3061: 2008 stated the density range for black pepper seeds oil as 0.861 to 0.885 g/mL, and black pepper seeds and stem essential oils of this study were measured in the specific range, while leaves essential oil was a bit higher (0.890 g/mL). The refractive index can be defined as the ratio of the sine of the refraction angle when light passes from different media and represents a characteristic physical constant of an oil (Barak et al. 2023). The three essential oils studied displayed higher refractive indexes than the ISO 3061:2008 standard (1.480 to 1.493) and black pepper seeds essential oil from Bangladesh (1.476) (Morshed et al. 2017). This might be due to the different components in the oils (Quoc 2021).

### 4.4 Antimicrobial Activity of Pepper Essential Oils

Among the three parts of the pepper plant, stem essential oil shows strong antibacterial activity, which was attributed to its more antibacterial active ingredients (sesquiterpenoids). In comparison, black pepper seeds and leaves pepper essential oils show lower antibacterial activity since their ingredients were less inclined to fight against *S. aureus* and *E. coli* . Similar to the previous study, sesquiterpenoids provide more antimicrobial activity (Masyita et al. 2022). The antibacterial activity of pepper essential oils against gram‐positive bacteria was better than that of gram‐negative. It has been reported in many studies that this phenomenon is due to their different structures (Kang et al. 2022; Milagres de Almeida et al. 2023; Permadi et al. 2025).

This pattern suggests that the antibacterial effects of pepper essential oils are closely linked to their specific chemical composition rather than simple differences among plant parts. Sesquiterpenoids such as *β*‐caryophyllene, *δ*‐elemene, and *α*‐selinene are known to integrate into bacterial membranes, disrupting their integrity and leading to leakage of vital intracellular contents (Masyita et al. 2022). The stronger inhibition of *S. aureus* compared with *E. coli* can be attributed to differences in cell wall structure, where the thick peptidoglycan layer of Gram‐positive bacteria is more vulnerable to hydrophobic essential oil components, while the outer membrane of Gram‐negative bacteria provides additional protection (Indriyani et al. 2024; Permadi et al. 2025). These findings indicate that the unique metabolite profile of stem oil underlies its superior antibacterial potency and highlight its potential as a natural antimicrobial resource.

### 4.5 Antioxidant Activity of Pepper Essential Oils

The DPPH assay indicated that the stem essential oil exhibited the highest antioxidant activity among the three samples (stem > leaf > seed); however, in absolute terms its performance still falls within a weak category when expressed by TEAC. This pattern agrees with reports that the antioxidant capacity of *P. nigrum* essential oils is modest and highly dependent on plant part, origin, and extraction conditions, with some studies finding different parts (e.g., seed or pericarp) outperforming others under specific protocols (e.g., hydrodistillation vs. microwave‐assisted) (cf. Lee et al. 2020; Kusumorini et al. 2022; Nurzaman et al. 2022). Our data therefore refine the current understanding by showing that, under our conditions, stem oil ranks highest but remains weak in DPPH terms.

The superior (though weak) antioxidant outcome for the stem oil is consistent with its sesquiterpene‐rich profile, notably *δ*‐elemene, *β*‐caryophyllene, and *α*‐selinene—which are lipophilic terpenoids capable of interacting with radical species and cell membranes; synergistic effects among these constituents likely contribute to the measured activity. In contrast, the seed and leaf oils, with higher proportions of monoterpenes such as *δ*‐3‐carene and limonene, showed comparatively lower scavenging capacity. Together with the PCA separation of samples, these results suggest that relative enrichment in specific sesquiterpenes, rather than total terpene content, better explains the observed differences in antioxidant behavior across plant parts.

## 5 Conclusion

Black pepper seed essential oil contained 20 compounds, stem oil contained 29, and leaf oil contained 19, with *δ*‐3‐carene, limonene, and *β*‐caryophyllene dominating the seed oil; *δ*‐elemene, *δ*‐3‐carene, and *β*‐caryophyllene in the leaf oil; and *α*‐selinene, *β*‐caryophyllene, and *δ*‐elemene in the stem oil. Beyond confirming known antibacterial properties of pepper oil, this study highlights distinct compositional profiles among seeds, leaves, and stems, which were clearly differentiated by their bioactive constituents. These compositional differences translated into variable antibacterial activities, with leaf oil being more effective against *E. coli* and stem oil showing the strongest inhibition of *S. aureus* . Antioxidant evaluation using the DPPH assay further revealed that stem oil possessed the highest radical scavenging capacity (IC_50_ 255.10 ppm; TEAC 8.39 μmol TE/g), followed by leaf oil (IC_50_ 358.62 ppm; TEAC 5.98 μmol TE/g) and seed oil (IC_50_ 485.98 ppm; TEAC 4.41 μmol TE/g), although all activities were relatively weak in absolute terms. Such findings not only extend the understanding of chemical diversity in different pepper plant parts but also demonstrate their potential as alternative, underutilized sources of bioactive essential oils with both antibacterial and antioxidant properties for food preservation and natural health applications.

## Author Contributions

**Sarifah Nurjanah:** conceptualization (lead), data curation (lead), writing – original draft (lead). **Edy Suryadi:** formal analysis (equal), investigation (equal). **Ahmad Thoriq:** resources (equal), software (equal). **Nurul Ainina:** formal analysis (equal), resources (equal). **Efri Mardawati:** data curation (equal), project administration (equal). **Muhammad Gilang Ramadhan:** visualization (equal). **Rosmiati Rosmiati:** formal analysis (equal), resources (equal). **Abd. Wahid Rizaldi Akili:** visualization (supporting), writing – review and editing (supporting). **Nandang Permadi:** visualization (supporting), writing – review and editing (supporting). **Euis Julaeha:** funding acquisition (equal), validation (equal).

## Conflicts of Interest

The authors declare no conflicts of interest.

## Supporting information


**Table S1:** List of significantly correlated variables to PC 1.
**Table S2:** List of significantly correlated variables to PC 2.

## Data Availability

Data is contained within the article.
