# Supplementary material for: Essential Oils From Different Parts of Piper nigrum L.: Chemical Composition, Antibacterial, and Antioxidant Activities
Source: Food Sci Nutr. 2025 Nov 14;13(11):e71205. doi: 10.1002/fsn3.71205 (PMC12618210; doi:10.1002/fsn3.71205)
Supplement: Supplementary file 1 — Table S1: List of significantly correlated variables to PC 1. Table S2: List of significantly correlated variables to PC 2. [file FSN3-13-e71205-s001.docx]

Table S1. List of significantly correlated variables to PC.1

| Compound | Correlation | *p*-value |
| --- | --- | --- |
| Humulen | 0.999534052 | 3.25 x 10^-7^ |
| germacrene.D | 0.998563791 | 3.09 x 10^-6^ |
| Ledene | 0.998367589 | 3.99 x 10^-6^ |
| Naphthalene | 0.998367589 | 3.99 x 10^-6^ |
| gamma.cadinene | 0.996512561 | 1.82 x 10^-5^ |
| gama.gurjunene | 0.996288359 | 2.06 x 10^-5^ |
| beta.chamigrene | 0.995740428 | 2.71 x 10^-5^ |
| delta.elemene | 0.994795817 | 4.05 x 10^-5^ |
| Alloaromadendrene | 0.99416463 | 5.09 x 10^-5^ |
| alpha.gurjunene | 0.993683422 | 5.97 x 10^-5^ |
| beta.selinene | 0.991878783 | 9.87 x 10^-5^ |
| Isospathulenol | 0.990304893 | 1.40 x 10^-4^ |
| alpha.cubebene | 0.98834658 | 2.03 x 10^-4^ |
| Pentalene | 0.977070449 | 7.83 x 10^-4^ |
| beta.elemene | 0.962674879 | 2.06 x 10^-3^ |
| delta.Cadinene | 0.89540221 | 0.01583886 |
| alpa.terpinolene | -0.821053631 | 0.045167612 |
| delta.3.carene | -0.863910309 | 0.026520388 |
| beta.caryophyllene | -0.928847333 | 7.41 x 10^-3^ |
| Benzene | -0.965010064 | 1.81 x 10^-3^ |
| beta.myrcene | -0.98223682 | 4.70 x 10^-4^ |

Table S2. List of significantly correlated variables to PC 2

| Compound | Correlation | *p*-value |
| --- | --- | --- |
| linalool | 0.9866377 | 2.66 x 10^-4^ |
| beta.pinene | 0.8600493 | 0.0280087607 |
| eucalyptol | 0.8465658 | 0.0335070161 |
| Valencene | 0.8462196 | 0.0336542755 |
